# Nestin expression in intact and hypertrophic myocardium of spontaneously hypertensive rats during aging

**DOI:** 10.1007/s10974-023-09641-9

**Published:** 2023-01-24

**Authors:** Dana Cizkova, Jitka M. Zurmanova, Lucie Gerykova, Alexandros Kouvelas, Mario Heles, Barbara Elsnicova, Frantisek Galatik, Jan Silhavy, Michal Pravenec, Jaroslav Mokry

**Affiliations:** 1 Department of Histology and Embryology, Faculty of Medicine in Hradec Kralove, Charles University, Hradec Kralove, Czech Republic; 2 Department of Physiology, Faculty of Science, Charles University, Prague, Czech Republic; 3 Institute of Physiology, Czech Academy of Sciences, Prague, Czech Republic

**Keywords:** Nestin, Desmin, Vimentin, Myocardium, Myocardial hypertrophy

## Abstract

Nestin is a unique intermediate filament expressed for a short period in the developing heart. It was also documented in several cell types of the adult myocardium under pathological conditions such as myocardial infarction or fibrosis. However, circumstances of nestin re-occurrence in the diseased or aging heart have not been elucidated yet. In this work we immunohistochemically detected nestin to determine its expression and distribution pattern in the left ventricular myocardium of normotensive Wistar Kyoto (WKY) rats and in the hypertrophic ones of spontaneously hypertensive (SHR) rats, both at the age of 1 and 1.5 year. No nestin^+^ cells were identified in the intact myocardium of 1-year-old WKY rats, whereas in the aged 1.5-year-old WKY rats nestin^+^ endothelial cells in some blood vessels were discovered. In the hypertrophic myocardium of all SHR rats, nestin was rarely detected in desmin^+^ vimentin^−^ cardiomyocytes and in some vimentin^+^ interstitial cells often accumulated in clusters, varying in intensity of desmin immunoreactivity. Moreover, nestin was infrequently expressed in the endothelial cells of some myocardial blood vessels in 1-year-old SHR rats, but not in 1.5-year-old ones. Quantitative image analysis of nestin expression in the myocardium confirmed significant increase in 1.5-year-old WKY rats and in SHR rats of both ages compared to the intact 1-year-old WKY rats. This study firstly documents nestin re-expression indicating cytoskeletal remodelling in different cell types of the aging intact and chronically pressure over-loaded hypertrophied myocardium. Our findings confirm nestin involvement in complex changes during myocardial hypertrophy and progressive aging.

## Introduction

Nestin (neuroepithelial stem cell protein) is an intriguing intermediate filament protein with unique properties, recently added to the class IV to neurofilaments and internexin (Bernal and Arranz 2018). Since its identification in neuroepithelial stem cells by Hockfield and McKay (1985), nestin has been proven to be expressed in several other types of the stem cells playing roles in their self-renewal and division (Lendahl 1990; Bernal and Arranz 2018). In some developing tissues nestin is detected in proliferating and migrating cells. In adulthood, nestin can re-express in these tissues in processes when specific developmental phases are repeated and cell cytoskeletal components need to be remodelled, such as physiological renewal, regeneration, healing, revascularization or tumorigenesis (Michalczyk and Ziman 2005; Bernal and Arranz 2018).

In the skeletal muscle, nestin is expressed just at the beginning of its development, in myotomal cells of mouse somites (Kachinsky et al., 1994). In proliferating myoblasts, nestin copolymerizes with vimentin that disappears during subsequent differentiation, and forms intermediate filaments with desmin in maturing myotubes (Sejersen and Lendahl 1993; Carlsson et al. 1999). In mature muscle fibres, nestin is present only in the sarcoplasm adjacent to the neuromuscular and myotendinous junctions (Carlsson et al. 1999; Vaittinen et al. 1999; Cizkova et al., 2009a). Nestin is also expressed during regeneration in newly formed myoblasts and myotubes, both extrafusal and intrafusal within the muscle spindles (Vaittinen et al. 2001; Cizkova et al., 2009a; Cizkova et al., 2009b). Therefore, nestin is considered a suitable marker of skeletal muscle regeneration (Cizkova et al., 2009a; Cizkova et al., 2009b). This protein also appears in immature skeletal muscle fibres in patients with Duchenne/Becker muscle dystrophy and myositis (Sjöberg et al. 1994), and in rhabdomyosarcoma cells in child patients (Kobayashi et al. 1998).

In myocardium, nestin is expressed between E9 and E10.5 in the developing mouse (Kachinsky et al., 1995). It has not been detected in cardiomyocytes in the adult intact heart (Mokry et al. 2008; Berry et al. 2013; Lionetti et al. 2014), however, its expression in the adult cardiomyocytes was determined under pathological conditions; after myocardial infarction in humans as well as in murine models, in human samples of the hearts affected by idiopathic dilated cardiomyopathy, in the heart of mouse model of the Duchenne/Becker muscle dystrophy and in the hypertrophic fibrotic heart of rat model of acute hypertension (Drapeau et al. 2005; Scobioala et al. 2008; Mokry et al. 2008; Berry et al. 2013; Lionetti et al. 2014; Hertig et al. 2017). In our previous work, we detected nestin in human myocardium of patients who died after myocardial infarction. In those samples, nestin^+^ cardiomyocytes were located in the vicinity to the necrotic area (Mokry et al. 2008), in some capillaries growing into the necrotic infarction area and infrequently in some interstitial cells. The presence of nestin in blood vessels in viable myocardium next to the infarction area gives evidence of increased angiogenesis in this region (El-Helou et al. 2005, 2008, 2013; Mokry et al. 2008; Yang et al. 2017). Moreover, Berry et al. (2013) and Hertig et al. (2017) described nestin immunoreactivity in interstitial cells of the adult myocardium.

For a long time, the myocardium has been considered having no regenerative capacity beyond early childhood, but findings of cardiac muscle stem cells indicate a possible regeneration potential (Messina et al. 2004; Tomita et al. 2005; Uchida et al. 2013). Since nestin is expressed in newly formed differentiating and maturing skeletal muscle cells, its appearance is undoubtedly related to skeletal muscle regeneration. An interesting question arises whether nestin^+^ cardiomyocytes and interstitial cells could participate in the process of the cardiac regeneration.

In this work we have studied the presence and distribution pattern of nestin and its co-assembling intermediate filaments desmin and vimentin in the myocardium of the aging spontaneously hypertensive rats (SHR) at the age of 1-year and 1.5-years manifesting heart failure phenotype, and age-matched normotensive Wistar Kyoto (WKY) rats. In SHR rats, severe and sustained systemic hypertension invariably occurs, accompanied by marked cardiac hypertrophy and interstitial proliferation. In contrast to many other experimental models, cardiac hypertrophy in SHR rats develops naturally and without any invasive interventions and proceeds chronically. Therefore, SHR rats represent a reliable model and experimental counterpart for essential hypertension and cardiac hypertrophy in man (Kawamura et al. 1976).

## Materials and methods

### Experimental animals

Two strains of adult male rats 1-year-old and 1.5-year-old were used in the present study. The Wistar Kyoto (WKY) rats were used as normotensive controls of an inbred spontaneously hypertensive rat strain SHR/OlaIpcv, which is characterized by hypertension and insulin resistance. Both rat strains were bred in the animal house of the Institute of Physiology, Czech Academy of Sciences. The rats were housed at a 12/12 h light-dark cycle and fed a standard diet.

### Histology

The hearts of the WKY rats (n = 5 per each group) and the SHR rats (n = 6 per each group) were excised under deep anaesthesia (Thiopental 60 mg/kg, i.p.), relaxed by perfusion on Langendorf apparatus with a Tyrode buffer and subsequently fixed with 4% paraformaldehyde in PBS as described previously (Waskova-Arnostova et al. 2015). The hearts were divided into several parts that were immersed in fresh 4% paraformaldehyde in PBS for next 48 h at room temperature, dehydrated and embedded in paraffin. Serial 6-µm-thick serial sections were cut from paraffin blocks using a microtome and every tenth slide was stained with haematoxylin-eosin for histological examination. To prepare cryosections, the heart samples were fixed by perfusion and subsequently immersed in 4% paraformaldehyde in PBS for 24 h at room temperature, soaked in 30% sucrose in PBS overnight at 4 °C, embedded in OCT (Tissue-Tek, Sakura) and snap-frozen in liquid nitrogen chilled isopentane (Sigma). Serial 10-µm-thick cryosections were cut using cryostat (Leica) and processed for immunohistochemistry.

### Immunohistochemistry and double immunofluorescence

Enzyme immunohistochemical detections were performed by indirect three-step LSAB method in cryosections or deparaffinised sections as described previously (Cizkova et al., 2018). Briefly, HistoStation (Milestone, Sorisole, Italy) was applied for antigen retrieval. Endogenous peroxidase was blocked in 5% H_2_O_2_ (3 × 10 min) and then, sections were incubated in 5% normal donkey serum (Jackson ImmunoResearch Laboratories, USA). Sections were incubated with primary antibody overnight at 4 ˚C and after washing in PBS, they were exposed to anti-rabbit or anti-mouse secondary biotinylated antibody (Jackson ImmunoResearch Laboratories, USA) for 45 min at room temperature. After rinsing, sections were incubated with streptavidin conjugated to horseradish peroxidase (DAKO) for 45 min and then the reaction was developed with 3,3-diaminobenzidine tetrahydrochloride (Sigma). Sections were dehydrated, counterstained with haematoxylin and mounted in DPX (Sigma).

For double immunofluorescent detections we have developed a novel method to enhance intensity of nestin signal based on application of Dako EnVision + System-HRP Labelled Polymer. Antigen retrieval was performed in HistoStation, sections were permeabilized in 0.5% Triton® X-100 (Sigma), endogenous peroxidase was blocked in 5% H_2_O_2_ (3 × 10 min) and 5% normal donkey serum (Jackson ImmunoResearch Laboratories, USA) was used to prevent unspecific binding. Then sections were incubated with mixture of the primary mouse anti-nestin and rabbit anti-desmin or rabbit anti-vimentin antibodies overnight at 4 ˚C. After thorough washing in PBS, sections were exposed to anti-mouse Dako EnVision + System-HRP Labelled Polymer (Dako) for 40 min at room temperature. Subsequently, following rinsing sections were incubated with mixture of the primary goat Cy3-conjugated anti-HRP antibody and secondary donkey anti-rabbit Cy2-conjugated antibody (both Jackson ImmunoResearch Laboratories, USA) for 45 min at room temperature. Sections were finally counterstained with DAPI (4,6-diamidino-2-phenylindole; Sigma).

The following primary antibodies were used: mouse monoclonal anti-nestin antibody, clone Rat-401 (Developmental Studies Hybridoma Bank, Iowa, USA; 1:4 or Millipore, 1:200), rabbit monoclonal anti-desmin antibody, clone RM234 (Genetex, 1:1500), rabbit monoclonal anti-desmin antibody, clone Y66 (Abcam, 1:250), rabbit monoclonal anti-vimentin antibody, clone EPR3776 (Abcam, 1:300).To avoid false immunopositivity, serial sections were processed according to the same protocol, but primary antibodies were omitted. Tissue sections were examined in Olympus BX51 microscope equipped with epifluorescence and DP71 camera.

### Nestin quantification

Custom-made automated macro in FIJI (Schindelin et al. 2012) was created for the analysis of nestin distribution on histological tissue slices. Two slices from each heart sample of all experimental groups (n = 5) comprising ten fields of view (FOV) from each slice were analysed. Nestin positive signal was expressed as an Area Fraction, i.e. the percentage of above-threshold pixels plotted using Color Threshold plugin in FIJI related to the overall tissue section. Data were analysed using GraphPad Prism (version 9.4.1., GraphPad Software). Data passed Shapiro-Wilk test for normal distribution, therefore the statistical evaluation was performed as Two-Way ANOVA with Sidak post-test for testing significant differences between animal strains of matching age and for testing the age effect in individual strains. The significance levels for each analysis are indicated in the Figure legends.

## Results

To reveal nestin expression and distribution in the normal and hypertensive aging heart, cardiac tissue samples were histologically evaluated using longitudinal and cross sections. The cardiomyocytes were identified by eosin staining and in longitudinal sections by cross striation pattern in their cytoplasm. Extent of fibrosis was qualitatively compared in Masson´s blue trichrome stained sections. Nestin+ cells were further chararacterized using nestin co-assebmling intermediate filaments either by tissue specific marker desmin and vimentin or by muscle cells development marker vimentin.

### Histological evaluation of normal and hypertensive aging heart

**Fig. 1:**
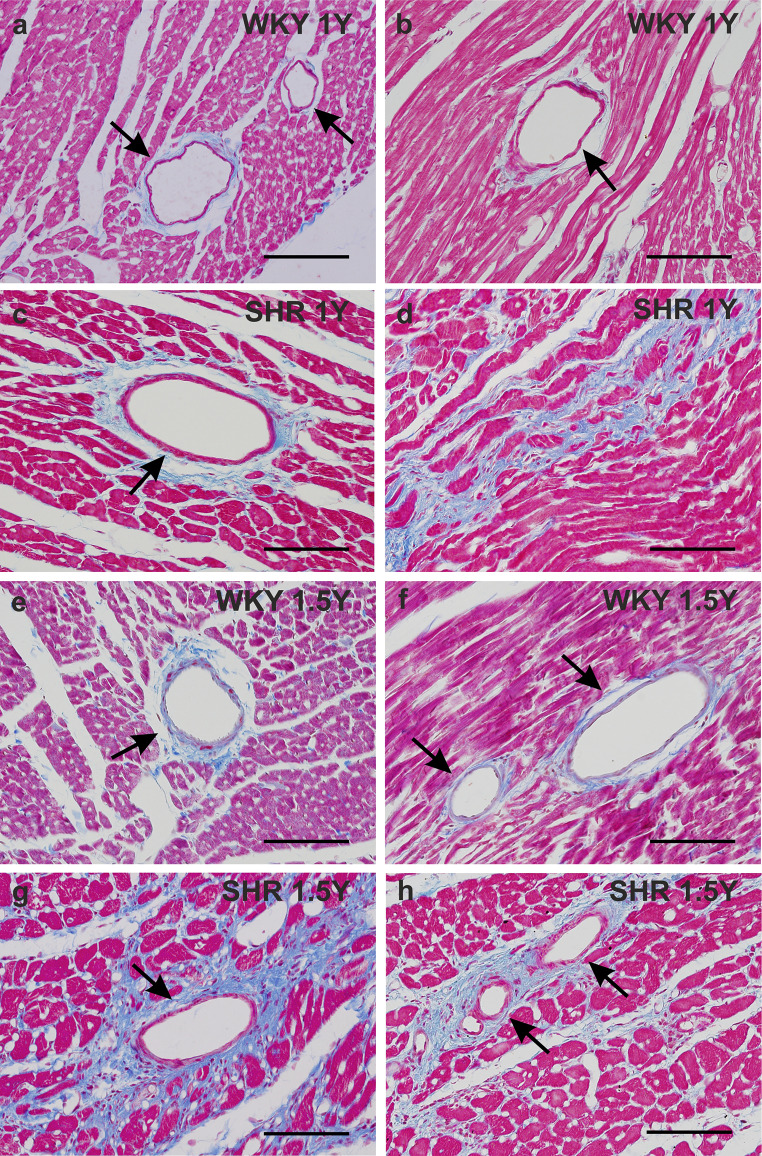
Histological structure of the myocardium of the 1-year-old control WKY rats (a, b) and SHR rats of the same age (c, d) and of the 1.5-year-old control WKY rats (e, f) and SHR rats of the same age (g, h). The cardiomyocytes in the intact 1-year-old WKY rats are of normal size (a, b, e, f), however, the cardiac muscle cells in the SHR rats have unambiguously increased their size, characteristically for hypertrophy, at both examined ages (c, d, g, h). The amount of the connective tissue (blue) around the blood vessels in the 1-year-old WKY rats is small, and only very slightly increased in the 1.5 -year-old ones (a, b, e, f arrows). In the heart of the SHR rats of both examined ages the amount of the connective tissue (blue) is obviously enlarged indicating myocardial fibrosis (c, d, g, h). The connective tissue (blue) is highly accumulated around the blood vessels (c, d, g, h arrows) and also appears among the cardiomyocytes (d, g). Blue trichrome staining. Bars a - h 200 μm

The cardiomyocytes of 1-year-old (Fig. 1 a,b) as well as 1.5-year-old (Fig. 1 e,f) WKY rats were of normal size and the amount of the connective tissue was only subtly increased in the older animals correspondingly to natural aging. In the hearts of 1-year-old SHR rats, the cardiomyocytes were unambiguously hypertrophic and the connective tissue became more abundant mainly around the blood vessels but also slightly among the cardiac muscle cells indicating progress of fibrosis (Fig. 1 c,d). The myocardium of 1.5-year-old SHR rats was formed by considerably hypertrophic cardiomyocytes and significant amount of the connective tissue not only around the blood vessels but also among the cardiac muscle cells, which signifies advanced fibrosis (Fig. 1 g,h).

### Nestin detection in normal and hypertensive aging heart

**Fig. 2:**
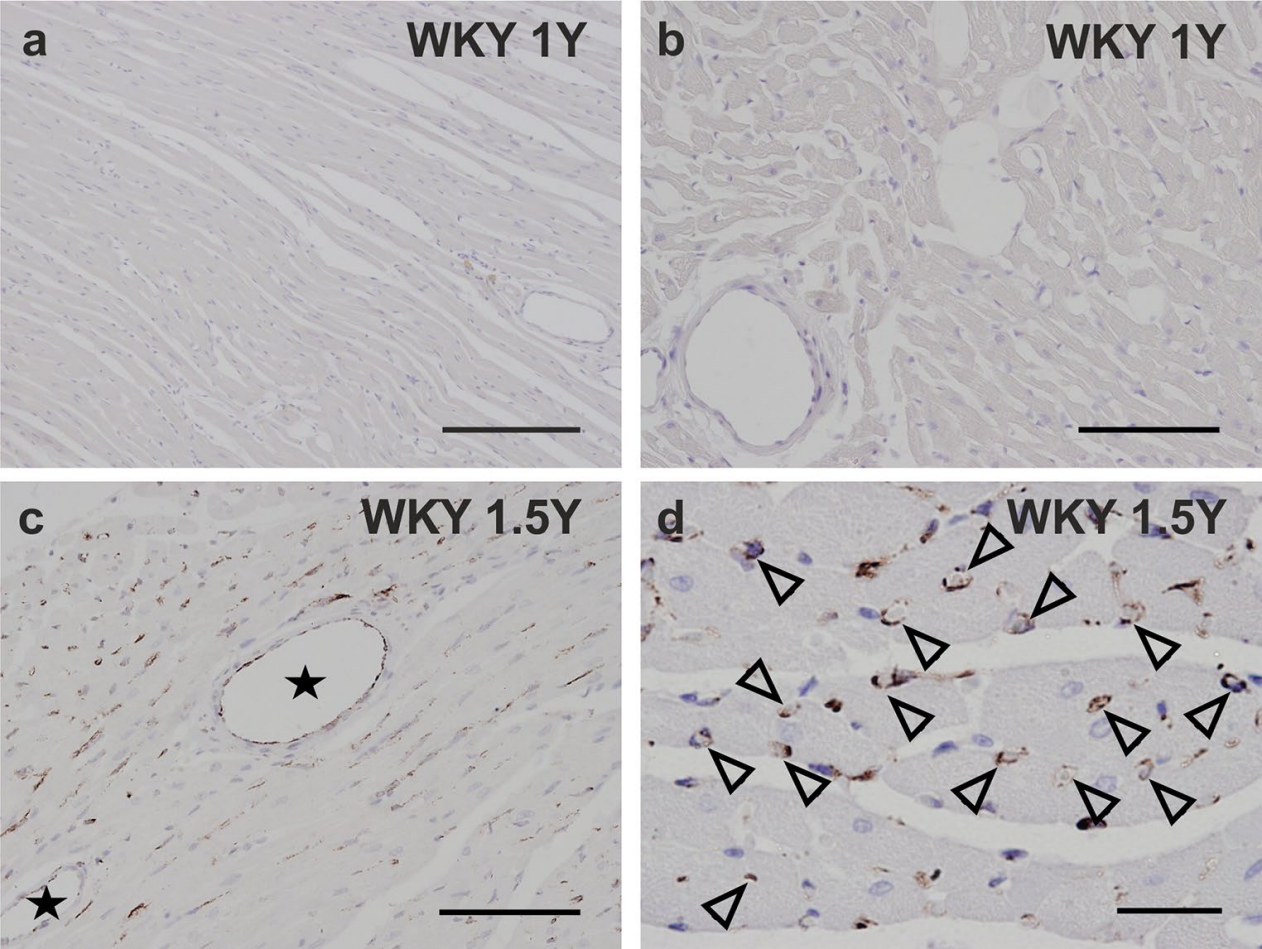
Immunohistochemical detection of nestin in the myocardium of WKY rats at the age of 1 year (a, b) and of 1.5 year (c, d). In the myocardium of the 1-year-old WKY rats nestin is not detected (a, b), while the 1.5 -year-old WKY rats nestin immunopositivity is documented in some endothelial cells of the blood vessels (c asterisks) and in some myocardial capillaries (d “empty” arrowheads), whereas the cardiomyocytes and the interstitial cells are nestin negative (c, d). Bars a 400 μm, b, c 200 μm, d 50 μm

**Fig. 3:**
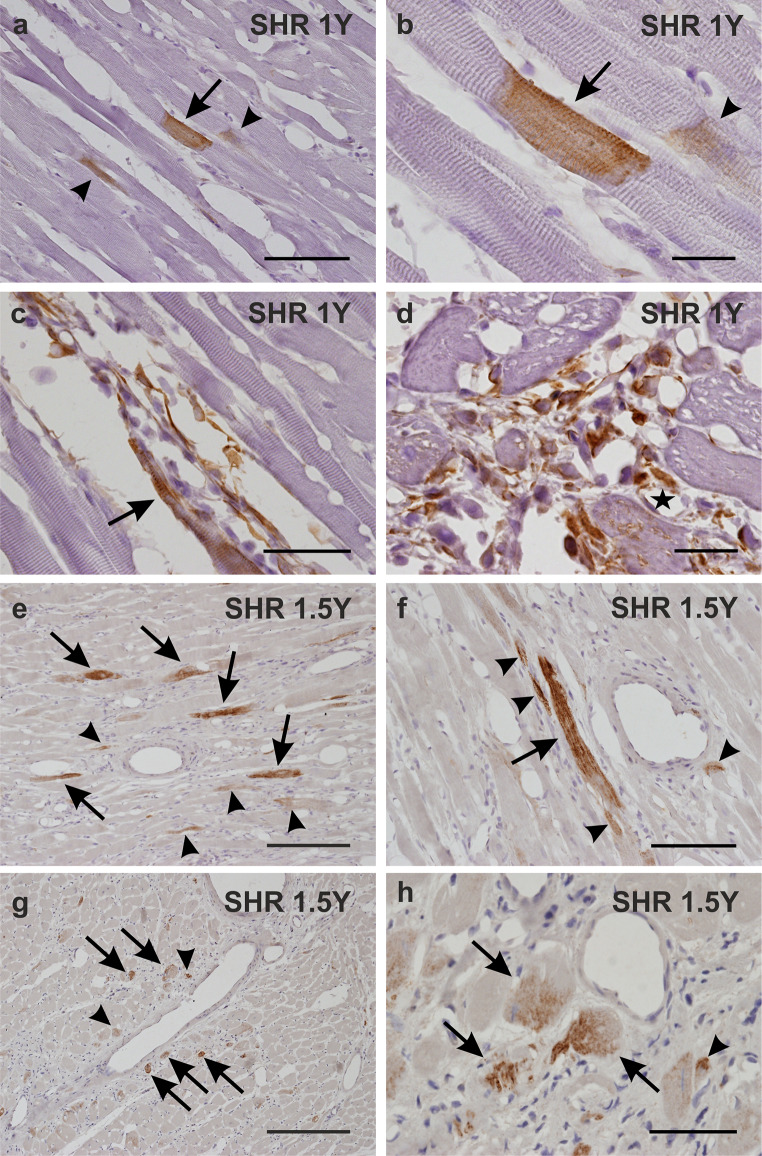
Immunohistochemical detection of nestin in the myocardium of the SHR rats at the age of 1 year (a, b, c, d) and of 1.5 year (e, f, g, h). In the myocardium of both SHR groups, nestin is detected in rare cardiomyocytes (a, b, e, f, g, h arrows). Using higher magnification characteristic cross striation in nestin+ cardiomyocytes is visible in longitudinal sections (a, b arrows). Nestin immunoreactivity is observed only in part of the cytoplasm in some cardiac muscle cells and sometimes in cardiomyocytes of smaller diameter compared to predominant nestin negative muscle cells (a, b, e, f, g, h arrowheads). Nestin is detected in some interstitial cells accumulated in groups located in areas formed by larger amount of the connective tissue (c, d). They are small and elongated with long projections or with oval body and short projections (c, d). A nestin+ cardiomyocyte of smaller diameter is noticed in the close vicinity to the nestin expressing interstitial cells (c arrow). Extremely rarely nestin is detected in the endothelial cells of the capillaries (d asterisk). Counterstained with haematoxylin. Cryosections a – d, deparaffinized sections e – h. Bars e, g 400 μm, a, f 200 μm, c, h 100 μm, b, d 50 μm

**Fig. 4:**
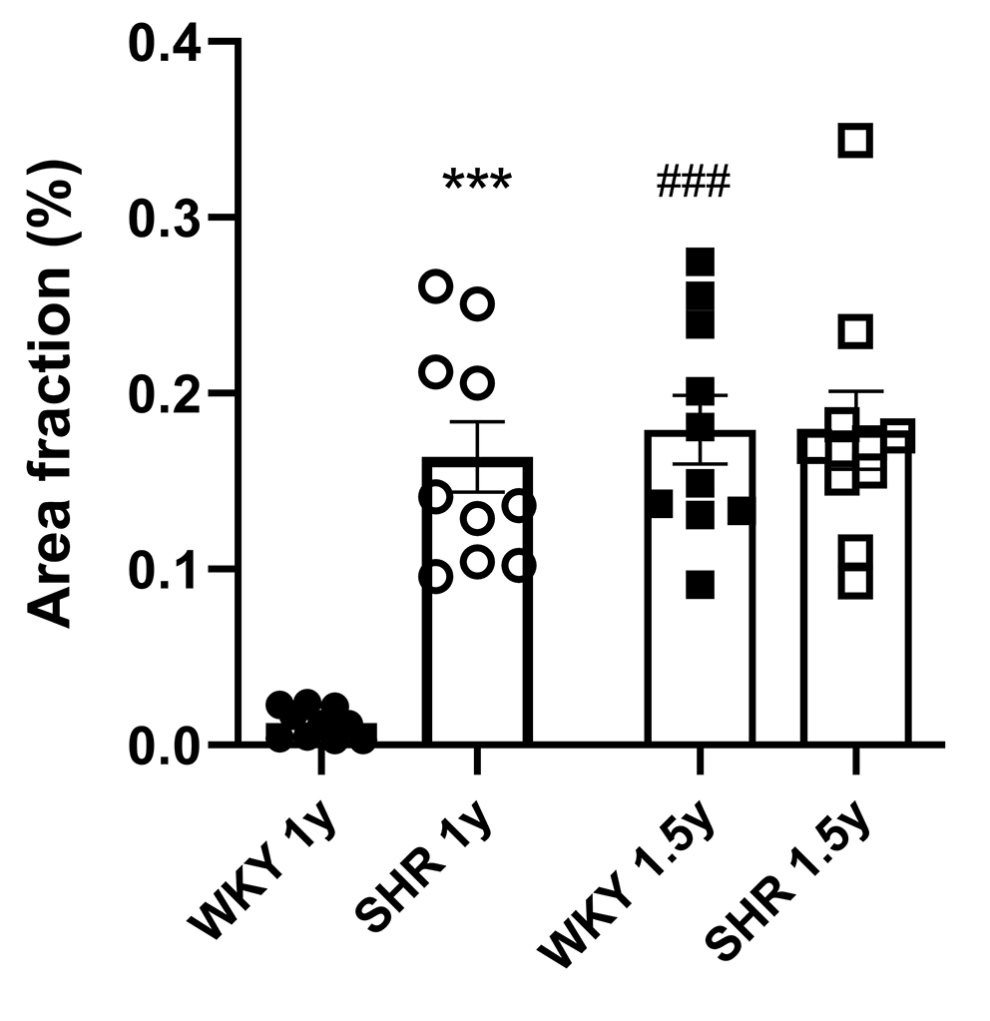
Quantification of nestin abundance and distribution on histological tissue slices of normotensive Wistar Kyoto controls (WKY) and inbred spontaneously hypertensive rats (SHR) was tested using Two-Way ANOVA with Sidák post-test. Analyses revealed significant differences between given strains at the age of 1 year but not at the age of 1.5 year; *** p < 0.001. The effect of aging (1 year vs. 1.5 year) was corroborated in the WKY strain only; ###p < 0.001. Presented data encompass two slices from each of 5 heart samples of each experimental group comprising ten fields of view (FOV) from each slice. Data points represent slice means, values are given as means ± SEM.

While nestin was not detected in any cells in the myocardium of the left ventricle and the septum in normotensive 1-year-old WKY rats (Fig. 2 a,b), in the 1-year-old SHR rats, nestin immunoreactivity was identified in some cardiomyocytes (Fig. 3 a-c). Nestin was also rarely detected in the connective tissue cells (Fig. 3 c,d) and in the endothelial cells of some blood vessels, mainly of capillaries (Fig. 3 d) in the 1-year-old SHR rats. The image analysis confirmed the significant difference in nestin expression represented as area fraction (in %) between 1-year-old WKY rats versus 1-year-old SHR rats (Fig. 4).

Concerning 1.5-year-old WKY and SHR animals, nestin was rare but present in both groups (Fig. 4) with a different morphological pattern. Detail analyses uncovered the presence of nestin exclusively within endothelial cells of capillaries in myocardium of the left ventricle and the septum in the normotensive WKY rats (Fig. 2 c,d), which may reflect the natural angiogenic proliferation in the normal aging heart. In the left ventricle and the septum of the hypertensive 1.5-year-old SHR animals, nestin was detected predominantly in some cardiomyocytes and in several interstitial cells, but importantly, not in the endothelium of capillaries as was determined using both deparaffinized longitudinal (Fig. 3 e-f) and cross (Fig. 3 g-h) sections. The quantification of nestin expression in the 1 and 1.5-year old SHR myocardium, expressed as area fraction (in %), did not show any significant differences (Fig. 4). These findings show a substantial difference in nestin spatial expression in the normal aging heart of WKY and pressure-overloaded aging heart of SHR rats.

In all SHR rats, nestin immunopositivity showing cross striation pattern in the cytoplasm confirmed cardiomyocyte phenotype (Fig. 3 a,b,f). Nestin^+^ cardiomyocytes were not located in any specific regions of the myocardium and majority of them did not differ in range of size from predominant nestin^−^ muscle cells, however, some nestin^+^ cardiac muscle cells of smaller diameter were also noticed (Fig. 3 a,b,e,f,g,h). In cross sections of some SHR cardiomyocytes a specific distribution of nestin signal was observed. It had the highest intensity at one site at the periphery of the muscle cell cytoplasm and the intensity decreased toward to opposite part of the cell (Fig. 3 g,h).

In both, the 1- and 1.5-year-old SHR rats, nestin immunoreactivity was rarely also noticed in interstitial cells accumulated in areas formed by a larger amount of the connective tissue whereas other cells within those groups were devoid of nestin signal. Nestin^+^ interstitial cells were relatively small and appeared elongated with long projections or with oval bodies and short projections (Fig. 3 c,d).

### Nestin colocalization with desmin and vimentin

**Fig. 5:**
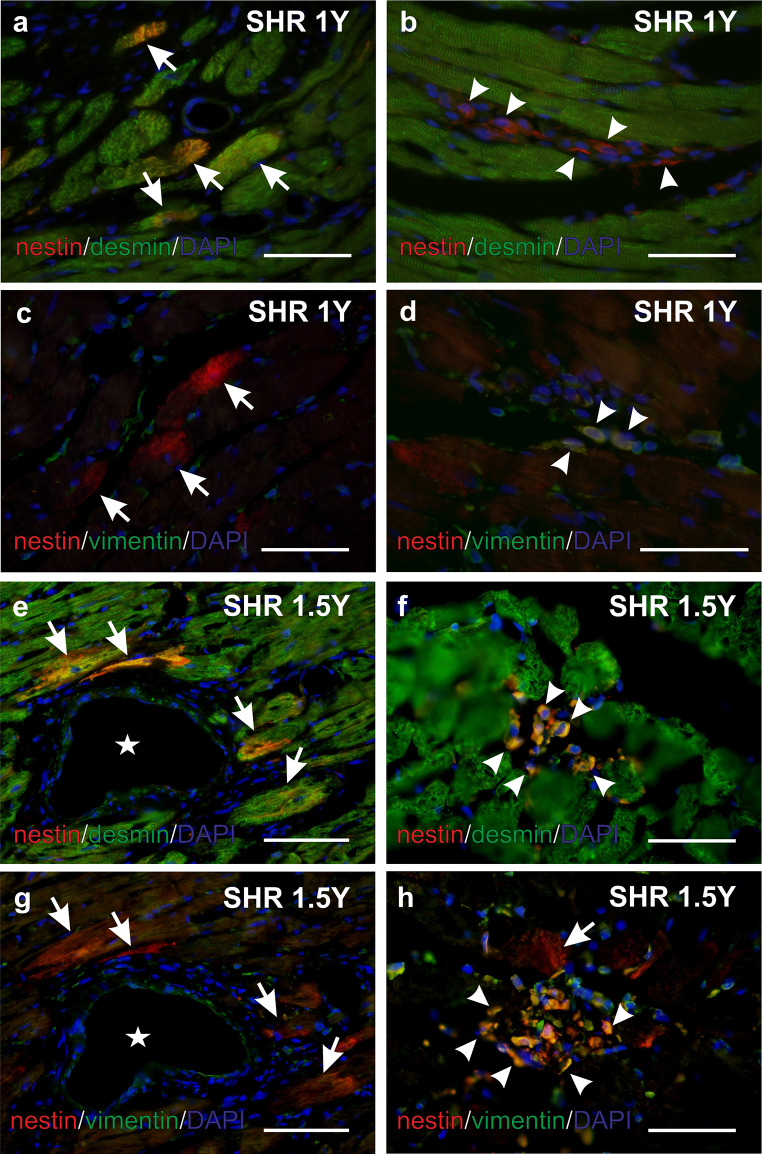
Immunofluorescent detection of nestin (red), desmin (green in a, b, e, f) and vimentin (green in c, d, g, h) in the myocardium of the 1-year-old (a, b, c, d) and 1.5-year-old (e, f, g, h) SHR rats. At both examined ages of SHR rats nestin is detected in rare desmin+ vimentin- cardiomyocytes (a, c, e, g arrows). Representative serial sections (e, g) shows the same region of the myocardium containing the vein (asterisk). Nestin is also detected in vimentin+ interstitial cells usually forming groups within the connective tissue (d, h arrowheads). These nestin+ interstitial cells differ in the intensity of desmin immunoreactivity regardless the age of the SHR rats (b, f arrowheads). The nestin+ interstitial cells depicted in the figure b (arrowheads) are desmin- , whereas in the Fig. 4f (arrowheads) they are desmin+ . Counterstained with DAPI (blue). Deparaffinized sections a – d, cryosections e – h. Bars e, g 200 μm, a-d, f, h 100 μm

In both WKY groups desmin immunoreactivity was observed in all cardiomyocytes and smooth muscle cells in the blood vessels wall (data not shown). Similarly, in both 1- and 1.5-year-old SHR rats, desmin was also detected in all cardiomyocytes and in the smooth muscle cells in the blood vessels wall. But moreover, in 1- and 1.5-year-old SHR rats, some interstitial cells mainly accumulated in areas formed by a larger amount of the connective tissue manifested desmin immunopositivity of different intensity (Fig. 5 a,b,e,f).

Vimentin was proved to be present only in the interstitial fibroblasts and in the endothelia and fibroblasts located in the wall of the blood vessels in WKY (data not shown) and SHR rat hearts.

Most nestin^+^ interstitial cells, forming clusters in the connective tissue of both SHR heart samples, were also vimentin immunoreactive though vimentin was not detected in any cardiomyocytes (Fig. 5 c,d,g,h). Notably, nestin^+^ cardiomyocytes of SHR samples carried desmin immunoreactivity, but no vimentin signal. In nestin^+^ interstitial cells vimentin was detected in majority of them whereas desmin only in some of them, with varying intensity of the signal (Fig. 5).

## Discussion

This is the first study documenting the expression of intermediate filament nestin in the chronically hypertrophied myocardium of aging spontaneously hypertensive rats. Importantly, in the hypertrophic myocardium, nestin was detected in rare desmin^+^ vimentin^−^ cardiomyocytes, and moreover, in some vimentin^+^ interstitial cells with different intensities of desmin immunoreactivity mostly accumulated in clusters in both, the 1-year-old and 1.5-year-old SHR rats. Nestin expression was also confirmed in some myocardial blood vessels endothelial cells in the 1-year-old SHR rats and surprisingly, also in the 1.5-year-old WKY rat hearts. Except for this finding, no other nestin^+^ cells were found in the normal myocardium of the WKY rats.

The SHR rats represent a model of essential hypertension in which the metabolic syndrome is also developed (Hajri et al. 2001; Cowley et al., 2006; Pravenec et al. 2014). The SHR inbred rat strain was established by repeated breeding of the inbred individuals of the WKY strain rats that showed spontaneous hypertension (Okamoto and Aoki 1963). In SHR rats there is a correlation between the increasing age and the degree of hypertension that is induced by excessive activity of the sympathetic nerve system, increased contractile power and increased sensitivity of the vascular smooth muscle to agonists (Okamoto and Aoki 1963; Pinterova et al., 2010). The hypertrophy of the myocardium begins to be apparent in 11 to 15-week-old SHR rats when focal interstitial fibrosis also occurs. In 1-year-old SHR rats, the cardiomyocytes are markedly hypertrophied in accordance with progress of the sustained hypertension and interstitial fibrosis increase in extent (Kawamura et al. 1976). In adulthood, the SHR rats show increased sensitivity to ischemia in comparison with the WKY rats, and in the age of 18 to 24 months they die of heart failure caused by cardiac fibrosis and decreased contractile function (Besik et al.,^2007^; Jullig et al., 2008).

### Nestin expression in cardiomyocytes

As a novel finding, we immunohistochemically confirmed nestin in the rare adult desmin^+^ vimentin^−^ cardiomyocytes in the SHR rat hearts while no nestin^+^ muscle cells were noticed in the control WKY rat hearts. The only published rat model of hypertension describing nestin positivity in cardiomyocytes concerns the suprarenal abdominal aorta constriction (SAC). In this case the nestin immunoreactivity was observed only in a modest population of the adult cardiomyocytes, however it was supposed to be secondary to ectopic ischemic injury associated to the concentric remodelling (Hertig et al. 2017).

Using the murine model, Meus et al. (2017) documented nestin^+^ cardiomyocytes situated in the peri-infarction region. Despite the characteristic appearance of nestin in proliferating cells and in an agreement with our previous findings of rare co-expression of nestin and PCNA in human periinfarction myocardium, those cardiomyocytes did not incorporated BrdU or co-expressed Ki67 (Mokry et al. 2008). Interestingly, nestin^+^ cardiomyocytes were noticed up to 28 days post-infarction in human patients and even 9 months in the rat model of the myocardial infarction (Mokry et al. 2008; Béguin et al., 2009). Scobioala et al. (2008) also detected nestin in some cardiomyocytes in the border zone surrounding the infarct area in the murine model. They also documented increased nestin mRNA levels in hearts from patients with acute myocardial infarction and chronic heart failure. In this context our 1.5-year-old SHR rats can be accepted as a model of the failing heart.

Lionetti et al. (2014) compared samples of the human failing hearts affected by the ischemic cardiomyopathy and idiopathic dilated cardiomyopathy with the intact myocardium and identified nestin^+^ cardiomyocytes in the diseased samples only. In the murine model of the Duchenne muscular dystrophy (the dystrophin/utrophin-deficient mouse) large clusters of nestin^+^ striated cells co-expressing cardiac troponin I, desmin and connexin 43, were present in the hearts near the end stage of the disease (Berry et al. 2013). Nestin^+^ cells revealing striations and elongated processes that expressed desmin but not cardiac troponin I were also noticed. Since cardiac troponin I represents a late cardiac marker, these muscle cells were found to be less matured than troponin I^+^ cells (Berry et al. 2013). To summarize, these findings give evidence that occurrence of nestin in some cardiomyocytes is related to the pathological changes in the myocardium including the myocardial hypertrophy.

### Nestin expression in interstitial cells

In the SAC rat model of the hypertension, nestin was detected in a subpopulation of the interstitial mesenchymal cells positive for collagen type I and negative for smooth muscle α-actin that were documented not only in the hearts of the hypertensive rats but infrequently also of the sham-operated animals. The authors considered these cells to be resident ventricular fibroblasts and nestin, rather than smooth muscle α-actin, to be a marker of an activated phenotype during the progression of the reactive fibrosis in the myocardium of the pressure-overloaded rats (Hertig et al. 2017). In our work we detected nestin in some interstitial cells that expressed vimentin and that manifested varying immunoreactivity of desmin. Such interstitial cells were detected in the hearts of the 1-year-old as well as 1.5-year-old SHR rats; Vimentin is usually present in cells of the mesenchymal origin such as fibroblasts but it is also expressed during the earliest stages of cardiogenesis. Since nestin occurs in the developing mouse cardiomyocytes between E9 and E10.5, it can copolymerize not only with desmin that appears on E8, but also with vimentin expressed at E8.5 – E9.5 (Schaart et al. 1989; Kachinsky et al., 1995). In our previous work, we documented the appearance of nestin^+^ interstitial cells in the border zone of some infarcted myocardial samples of human patients (Mokry et al. 2008). In peri-infarction area of the mouse heart nestin^+^ interstitial cells were found co-expressing some of four stem cell markers; c-kit, MDR1, Sca-1 and Abcg2. Moreover, Nkx2.5, a transcription factor restricted to the initial phase of cardiomyocyte differentiation, colocalized with nestin in certain cells. Proliferating cell nuclear antigen (PCNA) indicating proliferation activity of these cells, was detected in some nestin^+^ cells as well (Scobioala et al. 2008). In the dystrophin/utrophin-deficient mice, nestin^+^ interstitial cells were also noticed and majority of them expressed Flk-1 (VEGF Receptor 2). Flk-1 is a surface marker on the cardiac stem cells with potential to differentiate into cardiomyocytes, smooth muscle cells or endothelial cells (Berry et al. 2013). These findings may indicate spontaneous activation of resident stem cells leading to the production of progenitor cells and their differentiation into myocardial cells.

### Nestin expression in blood vessels

Nestin immunoreactivity was documented in some CD31^+^ endothelial cells of blood vessels in the left ventricle of the sham and pressure-overloaded SAC rats (Hertig et al. 2017). In the infarcted myocardium of the human patients nestin^+^ capillaries were found in the border zone, whereas the intact cardiac tissue was devoid of nestin immunoreactivity (Mokry et al. 2008; Scobioala et al. 2008) detected nestin in some endothelial cells and smooth muscle cells identified by von Willebrand factor or smooth muscle α-actin, respectively, in the border zone of the myocardial infarction in mice. In the non-infarcted area, the proportion of such cells was significantly smaller and in the heart of the sham-operated mice nestin^+^ vessel cells were missing (Scobioala et al. 2008). In the hearts of dystrophin/utrophin-deficient mice nestin^+^ endothelial cells were not found neither they were documented in the walls of the vessels in wild-type cardiac muscle (Berry et al. 2013).

Currently, nestin has been considered as a reliable marker of newly formed endothelial cells of the human as well as rodent blood vessels expressed during angiogenesis under physiological or pathological conditions (Mokry et al., 2004; Matsuda et al., 2013). In SHR rats, angiogenesis is reduced and native angiogenic response to ischemia is impaired (Emanueli et al. 2001; Wang et al., 2004). It corresponds to our findings of rare nestin^+^ blood vessels endothelial cells of the 1-year-old SHR rat hearts and no detection of nestin in such cells in 1.5-year-old SHR hearts. On the contrary, angiogenesis is activated after the myocardial infarction that results in appearance of nestin positivity in the endothelial cells of the blood vessels growing in the border zone (Mokry et al. 2008; Scobioala et al. 2008). Occurrence of nestin^+^ endothelial cells in some blood vessels of 1.5-year-old control WKY rats represents a very interesting finding documenting possibility of angiogenic proliferation in the normal aging heart and deserves further investigation.

### Nestin perspectives in cardiac regeneration

The myocardium is a tissue with extremely limited ability to renew, only 0.5–1% of cardiomyocytes are renewed yearly in adult humans (Bergmann et al. 2009; Witman et al. 2020). Origin of the newly formed cardiac muscle cells has not been fully elucidated yet, though proliferation of the pre-existing cardiomyocytes and differentiation of the cardiac progenitors have been studied. Also, prior to cardiomyocyte division, forgoing dedifferentiation and foetal program restart, described for example in Meus et al. (2017), could be accompanied by nestin re-expression. In these processes cell cytoskeleton is remodelled and can be also accompanied by nestin appearance. In the pressure-overloaded cardiomyocytes of SHR, the cytoskeleton is remodelled as well, together with progress of the hypertrophy. Nevertheless, nestin was expressed in only rare cardiac muscle cells in the SHR model. Scobioala et al. (2008) and Berry et al. (2013) supposed that nestin^+^ interstitial cells could represent precursors capable of differentiation into the cardiomyocytes. In the hypertrophied myocardium of the SHR rats, we identified morphologically similar nestin^+^ interstitial cells and thus, it is tempting to speculate that they could be myocardial precursors as well. Their precise immune-phenotypization using cardiac development, stem cells or progenitor markers would determine their cell type.

## Conclusion

In conclusion, nestin re-expression either in some cardiomyocytes or in certain interstitial cells of the hypertrophied myocardium reflects complex structural changes in the pressure over-loaded hearts. Occurrence of nestin in the endothelial cells of the blood vessels demonstrates substantial role of angiogenesis during aging in the intact as well as in early hypertrophied myocardium. Our findings give evidence of important nestin involvement in the myocardial hypertrophy and progressive aging. Further studies are needed regarding the possible role of nestin in cardiac regeneration.
